# Phenotypes of Klotho

**DOI:** 10.18632/aging.102117

**Published:** 2019-07-20

**Authors:** Jyoti Misra Sen

**Affiliations:** 1 National Institute on Aging, National Institutes of Health, Baltimore, MD 21224, USA; 2 Department of Medicine, The Johns Hopkins University School of Medicine, Baltimore, MD 21287, USA

**Keywords:** Klotho, Cyp24a1, vitamin D, epigenetics

*Klotho* gene was identified by Kuro-o et al. as hypomorphic mutant allele [*kl/kl*] that decreased expression of Klotho protein leading to pre-mature aging phenotypes and dramatic shortening of lifespan [1]. By contrast, transgenic expression of Klotho enhanced lifespan [2]. Taken together with the observation that Klotho expression declines with normal aging in humans and mice [3], these studies revealed a compelling role for Klotho in aging.

*kl/kl*-driven aging phenotypes in C3H, BALB/c and 129 genetic backgrounds correlated with altered mineral and vitamin D metabolic pathways, culminating in high levels of serum vitamin D [4]. Highest levels of vitamin D in serum, 5 to 6-fold higher compared to age matched controls, occur in 2-week old mutant mice, which then stabilize at approximately 3-fold higher in mice that live past the next few weeks [4]. Much additional work has pointed to high levels of vitamin D as being critical for aging phenotypes in *kl/kl* mice [5]. Thus, high vitamin D levels driven by low Klotho expression mediate aging-related phenotypes in C3H, BALB/c and 129 mice. Surprisingly, transfer of the *kl/kl* allele to a pure C57BL/6J background [B6-*kl/kl*] shows amelioration of aging phenotypes despite significantly reduced Klotho expression [6]. Concomitantly, the levels of vitamin D in the serum are also normalized in B6-*kl/kl* mice [7], consistent with the notion that high vitamin D levels in the serum promote aging-related phenotypes when Klotho expression is reduced.

Vitamin D levels in the serum are controlled by balanced expression of two enzymes, Cyp24a1 and Cyp27b1, in the kidney. Furthermore, signaling mediated by Klotho and FGF-23 heterodimer binding to FGFR regulates expression of these two enzymes in the kidney [reviewed in 8]. These studies indicate that different levels of expression of Cyp24a1 and Cyp27b1could be responsible for maintenance of vitamin D levels in C57BL/6 mice. To study the mechanism involved in regulation of vitamin D levels in the serum, expression of *Cyp24a1* and *Cyp27b1* was compared in mouse strains susceptible to aging-related phenotypes [C3H, BALB/c and 129] and one that does not show these phenotypes [C57BL/6]. Expression of *Cyp27b1* was found to be comparable in all four strains. By contrast, basal expression of *Cyp24a1* was found to be significantly higher in kidneys of C57BL/6 mice compared to C3H, BALB/c and 129 mouse strains [7]. Because signaling by Klotho via FGFR has been shown to regulate Cyp24a1, this observation suggests that low basal expression of *Cyp24a1* in susceptible strains may sensitize them to aging-related phenotypes, by augmenting the importance of FGF-23 and Klotho dimer-dependent Cyp24a1 expression to maintain healthy balance of vitamin D in the serum. Therefore, when Klotho is not expressed, the levels of vitamin D in the serum rise to dangerous levels leading to aging-related phenotypes and pre-mature death.

**Figure 1:**
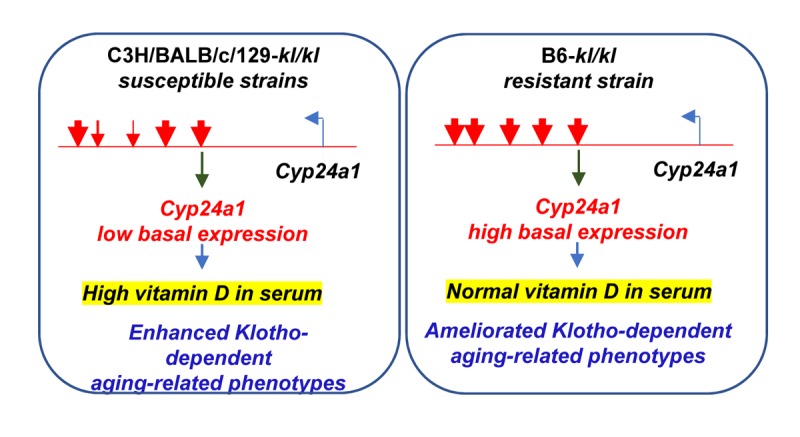
**Genetic variations in the enhancer-like regions downstream of the Cyp24a1 gene regulate its basal expression in different strains of inbred mice.** Deletions and substitutions in the putative *Cyp24a1* regulatory sequences in susceptible C3H, BALB/c and 129 mice lead to reduced basal expression of the gene, compared to resistant C57BL/6 mice, and render susceptible strains to high levels of vitamin D in the serum and aging-related detrimental phenotypes with decreased Klotho expression.

To address differential expression of Cyp24a1, chromatin structural features surrounding *Cyp24a1* gene were compared in C57BL/6 and susceptible C3H, BALB/c and 129 strains. Remarkably, super-enhancer like regulatory regions showed genetic variations including deletions and epigenetic alterations that were associated with lower expression of *Cyp24a1* in the kidney of C3H, BALB/c and 129 mice compared to C57BL/6 mice [7]. These observations suggest that genetic alterations in susceptible inbred strains, C3H, BALB/c and 129 result in lower basal expression of *Cyp24a1*, which renders the susceptible strains dependent on FGF-23/Klotho-mediated induced expression of *Cyp24a1* to regulate serum vitamin D levels. Significantly higher vitamin D levels in the serum promote aging-related phenotypes in the absence of support from Klotho/FGF23-dependent induced expression of *Cyp24a1* in susceptible genetic backgrounds [Figure 1].

Specifically, this work provides a plausible mechanism by which genetic background of inbred mice influence the impact of Klotho on aging-related phenotypes. Because of genetic diversity, the functional impact of Klotho levels in aging phenotypes in humans may also be differentially affected by vitamin D levels. Studies of Singh et al. [7] suggest that dysregulation of vitamin D metabolism may cooperate with alterations in Klotho expression to impact aging.
